# Integrative Analysis of Nutritional Components, Differential Metabolites, and Endophytic Microbiota Reveals Flavor Determinants of Lushan Russet Potato

**DOI:** 10.3390/foods15010067

**Published:** 2025-12-25

**Authors:** Libing Liao, Zhijun Yu, Yuhua Lu, Yihong Hu, Yushan Zhu, Yang Zhang, Deshui Yu

**Affiliations:** 1 Lushan Botanical Garden, Jiangxi Province and Chinese Academy of Sciences, Jiujiang 332900, China; liaolb@lsbg.cn (L.L.); zhijunyuls@163.com (Z.Y.); l2089232912@163.com (Y.L.); gzhuyihong@126.com (Y.H.); zhuysls@163.com (Y.Z.); 2 Jiangxi Provincial Key Laboratory of Wetland Plant Resources Conservation and Utilization, Jiujiang 332900, China; 3 Crops Research Institute of Jiangxi Academy of Agricultural Sciences, Nanchang 330200, China

**Keywords:** Lushan russet potato, nutritional compounds, metabolites, endophytic microbiome

## Abstract

The Lushan Russet potato, cultivated in Lushan Mountain (China), is renowned for its unique flavor, which deteriorates when cultivated at low altitudes. To unravel its flavor determinants, we compared high/low-altitude-cultivated Lushan Russet potato (LsM/LS) and reference Zhongshu5 (Zs) via nutritional, metabolomic, and endophytic microbiota analyses. LsM/LS had higher dry matter, potassium, and other flavor-related components than Zs. Non-targeted LC-MS metabolomics identified 461 metabolites. Pairwise comparisons revealed 263 significant differential metabolites (SDMs) between LsM and Zs (205 more abundant in LsM), 240 between LS and Zs, and 237 between LsM and LS. KEGG enrichment showed that SDMs were mainly involved in metabolic pathways. High-throughput sequencing of endophytic microbiota showed clear beta diversity separation, which correlated with metabolomic changes. These results indicate that Lushan Russet potato’s unique flavor is jointly determined by nutrient/metabolite accumulation and endophytic microbiome diversity, providing a basis for optimizing its quality and mitigating flavor deterioration in low-altitude cultivation.

## 1. Introduction

Potato plants (*Solanum tuberosum*) are cultivated worldwide, and China is one of the largest global producers and consumers of potatoes, producing almost 100 million tons of potatoes in 2022 (www.fao.org (accessed on 30 September 2024)). Potatoes are the world’s most vital non-cereal food crop, representing the primary source of carbohydrates and nutrients for millions of people [1,2]. There are thousands of potato varieties worldwide, which differ in potato tuber size, shape, color, starch content, and flavor [1]. Potatoes are considered a healthy food, as the tubers provide starch as well as dietary fiber, vitamins, and minerals (e.g., potassium, iron, calcium, zinc, sodium, and magnesium) [1,3,4]. Moreover, the contents of fat and sodium are low in potatoes and contain approximately 2% protein, with protein nutrition values compatible with those of egg protein [4]. Therefore, gaining insights into the factors regulating the accumulation of nutritional components and flavor-associated metabolites in potatoes is essential for optimizing tuber quality and meeting consumer demands for nutrient-rich, good-tasting food, which remains a central focus of research in the fields of agriculture and food science.

Metabolomics, a powerful approach to studying the complete metabolite set in biological materials, is crucial for deciphering the intricate links between food metabolites and their sensory properties [5,6]. This approach not only facilitates the identification of key flavor-active and aroma-contributing metabolites but also delivers profound insights into food quality formation, nutritional value, and potential health benefits, making it a cornerstone of modern food science research [7,8]. Metabolomics tools, particularly gas chromatography–mass spectrometry (GC-MS) and liquid chromatography–mass spectrometry (LC-MS), are commonly used to profile both volatile and non-volatile compounds in foods. Soluble compounds are often analyzed using LC-MS, whereas volatiles are typically identified with GC-MS [5,9]. Metabolomics can also offer a powerful approach to analyze the complex profile of flavor-related compounds, enabling the identification of metabolites responsible for distinct tastes [10,11]. However, research on potato metabolomics is relatively scarce; studies focusing on regionally distinctive potato cultivars—with unique flavor and phenotypic traits shaped by specific ecological environments—are even rarer.

Microbes play significant roles in influencing crop growth, yield, quality, nutritional content, and flavor through various mechanisms. For potatoes, soil microbial diversity directly correlates with tuber yield, starch accumulation, and nutrient content [12]. Under continuous cropping conditions, potatoes secrete nobiletin to recruit *Pantoea* sp. MCC16 strains, which promote root auxin (IAA) biosynthesis, increase adventitious roots, and enhance nutrient uptake, application of either the bacterium or nobiletin boosts yields by 35.86–51.09% [13]. Additionally, microbial consortia containing *Bacillus subtilis* and *Trichoderma harzianum* suppress soil-borne pathogens and enrich beneficial rhizospheric taxa, increasing potato yields by 23.0–32.2% [14]. Despite these advances, the specific correlations between microbial community structure and food quality traits (e.g., flavor, nutrient composition) remain largely elusive. Investigating these microbial–metabolic interactions is crucial for unraveling the biological mechanisms governing potato flavor formation and offers novel avenues for quality improvement.

The Lushan Russet potato, a geographically distinctive cultivar exclusively cultivated in the Lushan Mountain region of Jiujiang City, Jiangxi Province, China, has long been recognized for its exceptional organoleptic properties and morphologically unique russet peel—characterized by a rough, reticulated (net-like) surface texture that serves as a key phenotypic marker of this variety. Notably, when this cultivar is transplanted and grown in lower-altitude areas at the mountain foot, substantial alterations in its flavor profile have been observed. Furthermore, tubers harvested from these lower-altitude mountain foot environments exhibit a loss in seed viability, meaning they cannot be used as propagation material for subsequent cultivation cycles in the same lower-altitude conditions. This phenotypic and agronomic variation highlights the strong influence of environmental factors on the biological traits of the Lushan Russet potato, warranting systematic scientific investigation.

In this study, we analyzed the flavor profile of Lushan Russet potatoes grown in different positions and compared their nutritional components and metabolites with those of the Zhongshu 5 cultivar as a control. Further, we performed high-throughput DNA sequencing to analyze the endophytic microbiome diversity in Lushan Russet potato tubers. We aimed to understand the underlying causes of the distinct flavors and tastes of Lushan Russet potatoes grown in different positions when compared with the Zhongshu 5 potato.

## 2. Materials and Methods

### 2.1. Potato Plants

Lushan Russet potatoes were grown in an experimental field at the Lushan Botanical Garden on Lushan Mountain (hereafter abbreviated as LsM) at an altitude of approximately 1100 m (Jiujiang, China), or in an experimental field at the Poyang Lake Branch Garden of the Lushan Botanical Garden—located at the foot of Lushan Mountain (hereafter abbreviated as LS)—at an altitude of approximately 20 m. Zhongshu 5 potatoes (hereafter abbreviated as Zs) were grown in the same experimental field as LS (i.e., the Poyang Lake Branch Garden experimental field). Potato tubers were harvested approximately 4 months after seeding and initially stored at 4 °C for no more than 7 days in a dark, humidified incubator (Kesheng, Ningbo, China) (relative humidity: 65–70%) to minimize post-harvest metabolic fluctuations [15]. For all the analysis the tubers were rinsed extensively under running tap water for more than 4 h to remove surface soil. For the amplicon analysis of potato tubers, an additional set of treatments was performed. First, the tubers were soaked in 75% ethanol for 2 min to rapidly inactivate surface microorganisms. Subsequently, they were surface-sterilized by soaking in 2% sodium hypochlorite solution for 10 min, followed by more than 5 consecutive rinses with sterile ultrapure water to eliminate residual disinfectant and ensure the complete removal of epiphytic microorganisms’ contaminant. All samples were transported on dry ice for the analysis of nutritional components, non-targeted metabolite profiling, and endophytic microbial community assays. The peel of each potato was kept intact throughout the process. Each cleaned tuber was cut into small cubes (≈1 cm^3^), and a representative subsample was prepared by pooling pieces from the outer, middle, and inner regions of the tuber to eliminate spatial heterogeneity. The pooled subsample was immediately ground into a fine powder with liquid nitrogen for subsequent analysis.

### 2.2. Determination of Main Nutritional Components in Potato Tubers

The nutritional components of the potato tubers were determined by Wuhan ProNets Biotechnology Co, Ltd. (Wuhan, China). Three replicates were performed for each assay.

Sugar contents were determined using the anthrone colorimetric method described by Sun et al. [16]. The crude protein content was determined using the Kjeldahl method, according to a previous report [17]. Amino acid and crude fat contents were determined following the National Standards of the People’s Republic of China (NSC) GB/T8314-2013 and GB 5009.6-2016 guidelines, respectively. Potassium, calcium, magnesium, iron, and zinc contents were determined following the NSC-GB 5009.91-2017, NSC-GB 5009.92-2016, NSC-GB 5009.241-2017, NSC-GB 5009.90-2016, and GB 5009.14-2017 guidelines, respectively. The specific Limit of Detection (LOD) values are as follows: sugar (0.05 mg/g), free amino acids (0.05 mg/g), protein (0.04 g/100 g), potassium (K, 0.4 mg/100 g), sodium (Na, 1.6 mg/100 g), calcium (Ca, 1 mg/kg), magnesium (Mg, 2.4 mg/kg), zinc (Zn, 2 mg/kg), and iron (Fe, 1.5 mg/kg). For crude fat, LOD is not applicable.

### 2.3. Determination of Vitamin Content

Vitamin levels were measured using LC-MS assays. The levels of the fat-soluble vitamins A, D3, and E were determined following the NSC-GB 5009.82-2016 guideline.

The levels of the water-soluble vitamins B1, B2, B3, B6, B9, B12, and C (ascorbic acid) were determined according to a previously reported method with slight modifications [18]. Standard stock solutions of vitamins B1, B3, B6, B12, and C (500 μg/mL) were prepared using ultrapure water and temporarily stored at 4 °C. Before use, they were diluted with ultrapure water to concentrations of 2, 5, 10, 20, 50, 100, 200, and 500 μg/mL. For vitamin B2 and B9 solutions, 0.5% 1 M NaOH was used instead of water.

After grinding the samples into a fine powder with liquid nitrogen, approximately 0.5 g of each sample was weighed, extracted in the dark, thoroughly vortexed in 1 mL of ultrapure water, and sonicated for 30 min. Next, the samples were centrifuged at 11,000 rpm (12,851× *g*) for 5 min at 4 °C, and the supernatants were filtered through a 0.22 μm organic filter (Millipore, Burlington, MA, USA). A liquid chromatograph, LC-20AD, and a UV detector, SPD-M40 (both from Shimadzu, Kyoto, Japan), were used to record chromatograms and calculate the peak areas. Separation was conducted on an Athena C18 column (4.6 mm × 250 mm, 5 μm) with a mobile phase consisting of 0.1 M phosphoric acid solution (mobile phase A) and methanol (mobile phase B). Elution was conducted using the following gradients: 0.01 min, 95:5; at 8.00 min, 80:20; at 8.01 min, 45:55; at 16 min, 45:55; at 17.00 min, 95:5; at 28.00 min, 95:5. The flow rate was 0.5 mL/min, and the injection volume was 10 μL. Chromatograms were monitored at 245 nm, 256 nm, 287 nm, and 361 nm. LOD values of vitamins were determined as follows: vitamin C (VC), 0.01 μg/g; vitamin B_1_ (VB_1_), 0.01 μg/g; vitamin B_2_ (VB_2_, riboflavin), 0.05 μg/g; vitamin B_3_ (VB_3_, niacin), 0.01 μg/g; vitamin B_6_ (VB_6_), 0.01 μg/g; vitamin B_9_ (VB_9_, folic acid), 0.01 μg/g; vitamin B_12_ (VB_12_), 0.02 μg/g; vitamin A (VA), 0.2 μg/g; vitamin D (VD), 0.02 μg/g; and vitamin E (VE), 0.07 μg/g.

### 2.4. Metabolite Extraction

The extraction, identification, quantification, and analysis of non-targeted metabolites were performed by Personal Biotechnology Co., Ltd. (Shanghai, China). Six replicates were used in this assay. In brief, 200 mg of the sample was placed into a 2 mL centrifuge tube, and 600 µL of MeOH containing 2-amino-3-(2-chloro-phenyl)-propionic acid (at 4 mg/kg) was added. This mixture was vortexed for 30 s. Steel balls were then added, and the sample was placed in a tissue grinder TL-48R (Jingxin, Shanghai, China) for 60 s at 55 Hz and then underwent ultrasound for 15 min at room temperature. After centrifugation for 10 min at 12,000 rpm (15,294× *g*) and 4 °C, the supernatant was filtered through a 0.22 μm membrane and transferred into a detection bottle for LC-MS.

### 2.5. LC-MS

The sample extracts were analyzed using a Vanquish UHPLC System (Thermo Fisher Scientific, Waltham, MA, USA). Chromatography was performed using an ACQUITY UPLC^®^ HSS T3 (2.1 × 100 mm, 1.8 µm) (Waters, Milford, MA, USA). The column was maintained at 40 °C. The flow rate and injection volume were set to 0.3 mL/min and 2 μL, respectively. For LC-MS analysis, the mobile phase consisted of (B1) 0.1% formic acid in acetonitrile (*v*/*v*) and (A1) 0.1% formic acid in water (*v*/*v*). Separation was conducted under the following gradients: 0–1 min, 8% B1; 1–8 min, 8–98% B1; 8–10 min, 98% B1; 10–10.1 min, 98–8% B1; 10.1–12 min, 8% B1. For LC-ESI (−)-MS analysis, the analytes were carried out with (B2) acetonitrile and (A2) ammonium formate (5 mM). Separation was conducted under the following gradients: 0–1 min, 8% B2; 1–8 min, 8–98% B2; 8–10 min, 98% B2; 10–10.1 min, 98–8% B2; 10.1–12 min, 8% B2.

Mass spectrometric detection of metabolites was performed using an Orbitrap Exploris 120 (ThermoFisher Scientific, Waltham, MA, USA) with an ESI ion source. Simultaneous MS1 and MS/MS (full MS-ddMS2 mode, data-dependent MS/MS) acquisitions were used. The parameters were as follows: sheath gas pressure, 40 arb; aux gas flow, 10 arb; spray voltage, 3.50 kV and −2.50 kV for ESI(+) and ESI(−), respectively; capillary temperature, 325 °C; MS1 range, m/z 100–1000; MS1 resolving power, 60,000 FWHM; number of data-dependent scans per cycle, 4; MS/MS resolving power, 15,000 FWHM; normalized collision energy, 30%; dynamic exclusion time, automatic.

### 2.6. Data Preprocessing, Annotation and Multivariate Statistical Analysis

The off-machine raw data were extracted and converted to mzXML format using Proteowizard software (version 3.0). Raw peak identification, peak filtration, peak alignment, and internal standard normalization were performed. For multivariate analysis of the data, unsupervised principal component analysis (PCA) and orthogonal partial least squares discriminant analysis (OPLS-DA) were used. OPLS-DA was performed with 7-fold cross-validation (CV) to evaluate model overfitting, and the model quality was assessed by R^2^X (explained variance of the original data), R^2^Y (explained variance of the grouping variable), and Q^2^ (predictive ability). Significant differential metabolites (SDMs) were selected with a threshold *p*-value of ≤0.05 (Student’s *t*-test) and a variable importance in the projection (VIP) value of ≥1 (OPLS-DA) [19]. The metabolites were then annotated and identified based on public metabolome databases, such as the Human Metabolome Database (http://www.hmdb.ca (accessed on 10 August 2023)), Metlin (http://metlin.scripps.edu (accessed on 10 August 2024)), Massbank (http://www.massbank.jp (accessed on 10 August 2024)), LipidMaps (http://www.lipid-maps.org (accessed on 10 August 2024)), mzCloud (https://www.mzcloud.org (accessed on 10 August 2024)), MoNA (https://mona.fiehnlab.ucdavis.edu (accessed on 10 August 2024)), NIST_2020_MSMS spectral library, and the self-built standards database Personal Biotechnology Co., Ltd. (Shanghai, China). The MS1 mass tolerance was set to 15 ppm, and the MS2 Match Factor Threshold was set to 50. In this project, both the local self-built database and the public database were searched. The structural identification of metabolites in biological samples was performed by matching with information such as retention time, molecular mass (with a molecular mass error within <10 ppm), MS/MS fragmentation spectra, and collision energy of metabolites in the databases. Furthermore, the identification results were subjected to strict manual secondary verification and confirmation. The identification level was Level 2 or higher [20]. Moreover, the Kyoto Encyclopedia of Genes and Genomes (KEGG) (http://www.genome.jp/kegg/ (accessed on 10 August 2023)) and MetaboAnalyst 4.0 (http://www.metaboanalyst.ca (accessed on 10 August 2023)) were utilized to assess the metabolic pathways of the identified metabolites. Machine learning, other analyses, and most metabolome-related graphings were conducted using the Personal Gene Cloud (https://www.genescloud.cn (accessed on 24 April 2025)).

### 2.7. DNA Isolation and Amplicon Sequencing

DNA extraction was performed according to our previous study with 0.2 g of potato tuber; the DNA was stored at −20 °C. The bacterial 16S V3-V4 region was amplified using the primers DNA using the primers 341F and 805R (F-5′-CCTACGGGNGGCWGCAG-3′ and 5′-GACTACHVGGGTATCTAATCC-3′). The partial fungal ITS1 region was amplified using the primers ITS1F and ITS2R (5′-CTTGGTCATTTAGAGGAAGTAA-3′ and 5′-GCTGCGTTCTTCATCGATGC-3′). The samples were sequenced on an Illumina NovaSeq 6000 platform at the Wuhan Benagen Technology Company Limited (Wuhan, China) as described in our previous study [21].

### 2.8. Data Processing and Bioinformatic Analysis

Data processing and Bioinformatic analysis were performed as described in our previous study [21], except for the bacterial and fungal ASVs, which were assigned to taxonomic groups using the SILVA database (https://www.arb-silva.de/ (accessed on 2 September 2023)) and UNITE database (https://unite.ut.ee/ (accessed on 2 September 2023)) [22] for species annotation, respectively.

## 3. Results and Discussion

### 3.1. LsM/LS and Zs Potato Tubers Differ in Size and Shape

**Figure 1:**
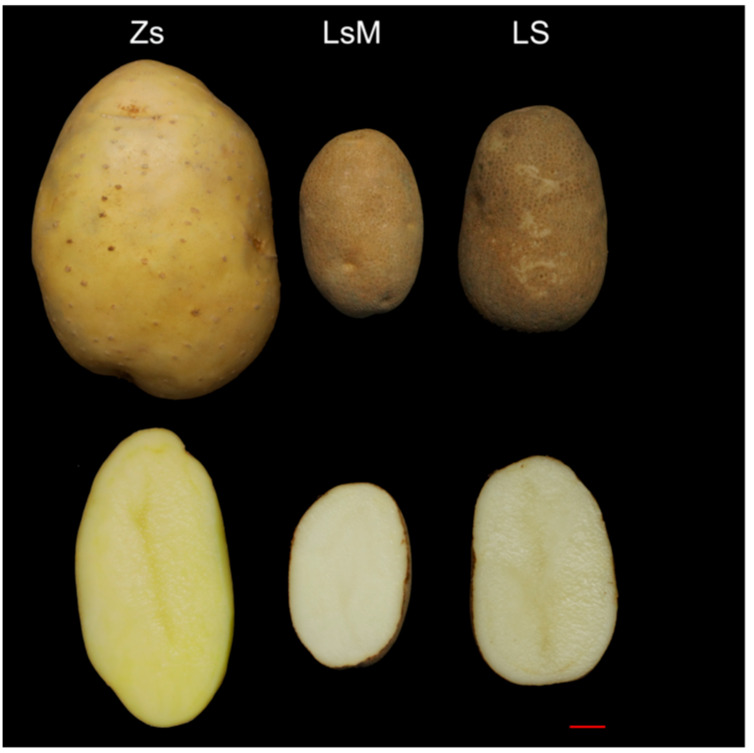
Morphological comparison of potato tubers among Zhongshu 5 (Zs), high-altitude Lushan Russet potato (LsM), and low-altitude Lushan Russet potato (LS). Upper panels, potato tubers (left, Zs; middle, LsM; right, LS). Lower panels, flesh of potato tubers (left, Zs; middle, LsM; right, LS). Scale bars: 1 cm.

Lushan Russet potato is a local specialty of the Lushan Mountain in China and is famous for its unique flavor, which attracts many tourists. Zhongshu 5 potato is a high-yield, early-maturing, disease-resistant, and widely planted potato cultivar developed from Zhongshu 3 in China, and was used as a reference potato in this study. The Lushan Russet potato (LsM/LS), also known as the Lushan small potato, exhibits characteristic oblong to elongated tubers with a distinctive russet skin, creamy white-colored flesh, and a very small size of approximately 4 cm in diameter and 6 cm in length (Figure 1 middle panels). When cultivated at lower altitudes, Lushan Russet potato tubers undergo morphological and flavor changes (Figure 1, right panels). Moreover, tubers harvested from lower elevations perform poorly as seed potatoes when replanted at the same altitude, often resulting in near-total yield failure. Conversely, Zhongshu 5 (Zs) tubers were slightly flattened and round and exhibited smooth yellow skin and light-yellow flesh (Figure 1, left panels). LsM and LS tubers are much smaller than Zs tubers (Figure 1), and thus have a low yield/unit. Additionally, the Lushan Mountain has limited available land, which contributes to a very low yield of LsM. As such, the potato’s price is very high, even exceeding the price of beef.

### 3.2. Nutritional Component Analysis

**Figure 2:**
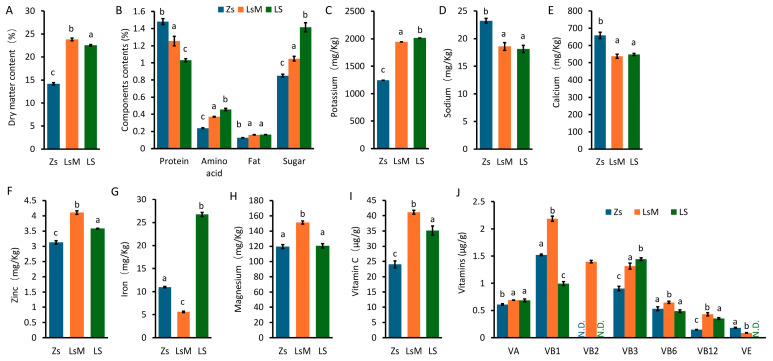
Nutritional components of Lushan Russet potato (LsM/LS) and Zhongshu 5 (Zs) potato tubers. (**A**) Contents of dry matter (DMC). (**B**) Protein, amino acids, fat, and sugar contents. (**C**–**H**) Mineral contents. (**I**,**J**) Vitamin contents. Error bars represent standard deviations from three independent samples, and lowercase letters (a, b, c) above bars indicate significant differences (*p* ≤ 0.05) among groups. N.D.: not detected.

The nutritional components of LsM, LS, and Zs tubers were analyzed. Dry matter content (DMC) indexes the potato quality, and starch is the main carbohydrate, which comprises about 80% (in weight) of the dry matter of tubers [3,23,24]. The DMC of LsM and LS tubers (23.8.0% and 22.6%, respectively) was significantly higher than that of Zs tubers (~14.0%) (Figure 2A), indicating that LsM and LS have higher DMC values than those of Zs. These data may suggest that LsM and LS may provide more nutrients than Zs (Figure 2A).

The DMC of potato tubers is influenced by the plant genotype, growth conditions, and storage after harvest [15,23,24,25,26,27]. Pretreating potato tubers with effective microorganisms before planting can increase DMC [28]. Potatoes with higher DMC and calcium content are more resistant to mechanical impacts and thus exhibit better tuber appearance and overall quality [27,29].

The biological value of potato protein is higher than that of any other heavily consumed plant protein, including soybeans, and is comparable with that of whole egg protein [4]. We analyzed the protein, amino acid, fat, and sugar contents of LsM, LS, and Zs tubers. The protein content of LsM and LS was lower than that of Zs, altogether ranging from 1.0% to 1.5% (Figure 2B). The contents of amino acids, fat, and sugar were significantly higher in LsM (0.37%, 0.16%, and 1.05%) and LS (0.46%, 0.16%, and 1.42%) than those in Zs (0.24%, 0.13%, and 0.85%) (Figure 2B). Amino acids, fat, and sugar are predominantly responsible for the flavor of potatoes [30,31], and thus the aforementioned differences in their content may contribute to the differences in flavor between the two cultivars. The fat content of potatoes is lower than that of other foods, while high-fat diets are associated with health risks, especially cardiovascular disease; therefore, the demand for reduced-fat dairy products is rapidly increasing [31]. Potatoes, with their low-fat content, well-balanced flavor, and health benefits, may become the optimal food choice.

Minerals and vitamins are important nutritional components for human health [32]. Potatoes are rich in potassium, and our data indicates that the potassium content of LsM (1945 mg/kg) and LS (2015 mg/kg) is higher than that of Zs (1243 mg/kg) (Figure 2C). Furthermore, LsM and LS exhibited lower contents of sodium and calcium (Figure 2D,E) and higher content of zinc (Figure 2F) compared with Zs. But for iron content, LS (27 mg/kg) and Zs (11 mg/kg) are higher than that of LsM (6 mg/kg) (Figure 2G). Magnesium content is highest in LsM, about 25% higher than that of LS and Zs (Figure 2H).

Potato tubers contained higher levels of vitamin C than other foods, and LsM and LS contained almost 2 and 1.5 fold vitamin C levels of those in Zs, respectively (Figure 2I). LsM also contained higher levels of vitamins A, B1, B2, B3, B6, and B12 compared with LS and Zs. Vitamin B2 was not even detected in Zs and LS potato tubers (Figure 2J). Conversely, Zs tubers contained slightly higher vitamin E levels compared with LsM tubers (Figure 2J). Vitamin D3 was not detected in any of the potato samples (LsM, LS, and Zs), as its concentration was below the LOD in this study.

Nutritional components, especially vitamin and mineral contents, vary and depend on numerous factors, such as plant genotype and growth conditions, as well as experimental methods, including the sample’s storage environment and duration, and the determination technique [33]. LsM and LS with the same genetic background cultured in different filed, are differ in nutrition profile. Zs potatoes and Lushan Russet potato (LsM and LS) differ significantly in genetic background and exhibit distinct nutritional profiles. Our results on the contents of vitamins and minerals slightly differ from previous reports [4]. This may be due to the storage of harvested potato tubers at 4 °C, which may have degraded certain vitamins and minerals. Nevertheless, the contents of vitamins and minerals significantly differed between LsM, LS, and Zs.

### 3.3. Metabolomics Assay Results Differed Between LsM, LS, and Zs

LC-MS is widely used for plant metabolomics analyses [6,8,34]. The Lushan Russet potato is famous for its good flavor when cultured in Lushan Mountain. In this study, we performed LC-MS-based non-targeted metabolomics analyses to characterize flavor-related compounds that could explain the unique flavor of Lushan Russet potato and the growth conditions’ contribution to potato flavor. In the positive (POS) and negative (NEG) ion modes, a total of 19,652 and 8509 effective peaks were detected, respectively (Supplementary Tables S1 and S2).

**Figure 3:**
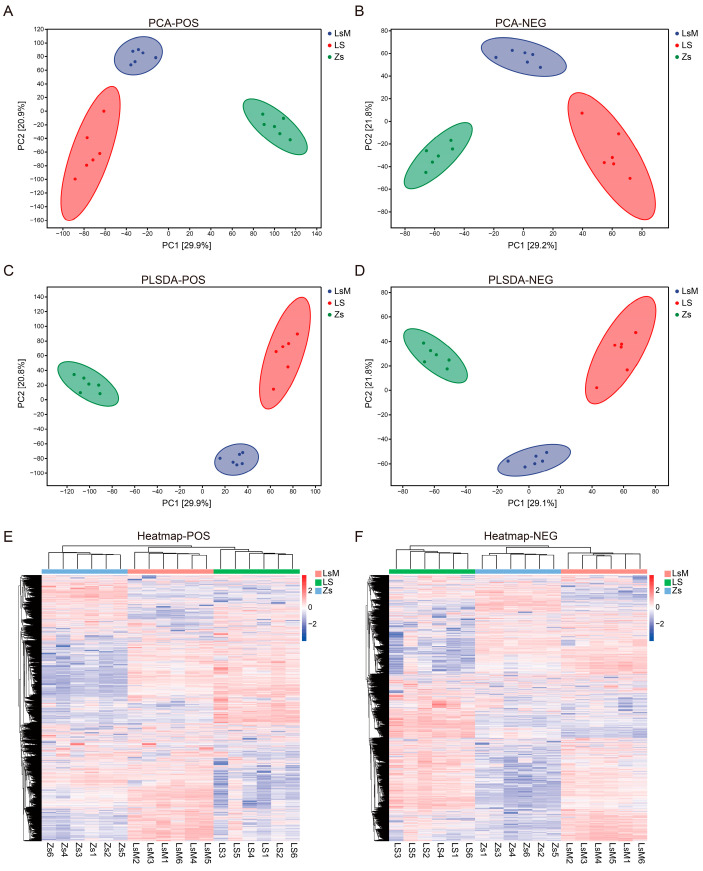
Different metabolic profiling of Lushan Russet potato tubers (LsM/LS) and Zhongshu 5 (Zs) potato tubers. (**A**,**B**) Principal component analysis using positive (**A**) and negative (**B**) ion mode data. (**C**,**D**) Partial least squares discriminant analysis (PLS-DA) using positive (**C**) and negative (**D**) ion mode data. (**E**,**F**) Heatmap analyses of positive (**E**) and negative (**F**) ion mode metabolic.

The effective peaks of the PCA and OPLS-DA results indicated a clear separation between the LsM, LS, and Zs samples, with the data points of each group tightly clustered (Figure 3A–D). The total contribution of the two principal components (PC1 and PC2) was about 51% for the total difference in metabolite concentration between LsM, LS, and Zs. For positive ion modes, PC1 and PC2 were 29.9% and 20.9%, respectively. For negative ion modes, PC1 and PC2 were 29.2% and 21.8%, respectively (Figure 3A,B). The OPLS-DA model components 1 and 2 explained 51.7% (Q^2^ = 0.956, R^2^X = 0.559, and R^2^Y = 0.993) and 50.9% (Q^2^ = 0.946, R^2^X = 0.562, and R^2^Y = 0.988) of the total differences between LsM, LS and Zs, for the positive and negative ion modes, respectively (Figure 3C,D). Cluster analysis was performed using all the effective peaks. Similar to the PCA and OPLS-DA results, samples were clustered into three groups according to the sample group, i.e., LsM, LS, and Zs, for both the positive and negative ion modes (Figure 3E,F).

Samples correlation analysis was performed using all metabolites, and the data showed that all the samples were highly correlated within groups but less correlated between LsM, LS, and Zs, which confirmed the results above (Supplementary Figure S1 and Supplementary Tables S3 and S4). Moreover, both the cluster heatmap (Figure 3E,F) and volcano plot (Supplementary Figure S2 and Supplementary Tables S5 and S6) showed that the metabolites with higher levels were more abundant in Lushan Russet potato tuber samples (LsM/LS) than in Zs samples (2296/3417 metabolites less abundant and 6372/5357 metabolites more abundant (POS) and 917/1086 metabolites less abundant and 2660/2769 metabolites more abundant (NEG)).

Notable intraspecific variations were observed among Lushan Russet potato accessions. Specifically, when compared with the LsM sample, the Ls samples exhibited differential metabolite profiles across two ionization modes: in the negative ion (NEG) mode, 1512 metabolites showed less abundant signals and 1765 metabolites displayed more abundant signals; in the positive ion (POS) mode, the number of metabolites with less abundant and more abundant signals was 4285 and 2644, respectively (Supplementary Figure S2; Supplementary Tables S5 and S6).

### 3.4. Identification of Significant Differential Metabolites (SDMs) Between LsM and Zs

The effective peaks from the LC-MS -based non-targeted metabolomics data were compared to public databases, such as the Human Metabolome Database (http://www.hmdb.ca (accessed on 10 August 2023)), Metlin (http://metlin.scripps.edu (accessed on 10 August 2023)), Massbank (http://www.massbank.jp (accessed on 10 August 2023)), and mzCloud (https://www.mzcloud.org (accessed on 10 August 2023)), which identified 447 and 147 metabolites in the positive and negative ion modes, respectively (Supplementary Table S7). In total, 334 metabolites in the positive and 127 metabolites in the negative ion modes belong to an identical class. The top abundance class consists of carboxylic acids and their derivatives, fatty acyls, steroids and their derivatives, organooxygen compounds, and benzene and its substituted derivatives. Of these, carboxylic acids and their derivatives comprise the most abundant metabolite class (Supplementary Table S7 and Supplementary Figure S3).

**Figure 4:**
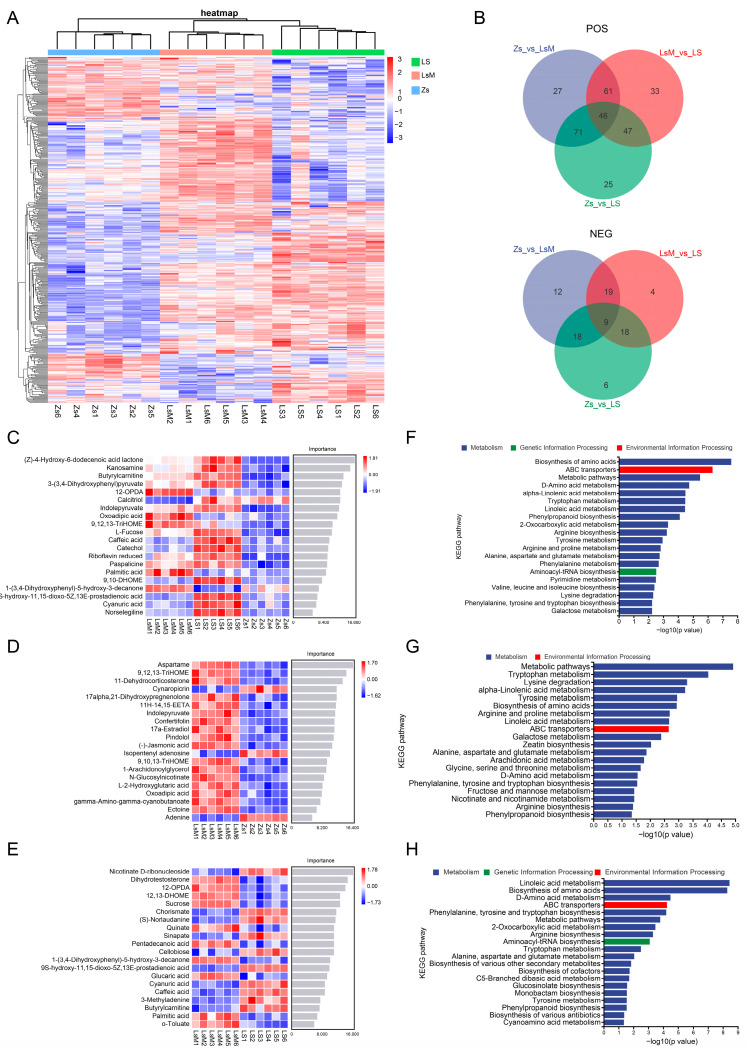
Significant differential metabolites (SDMs) between Lushan Russet potato tubers (LsM/LS) and Zhongshu 5 (Zs) samples. (**A**) Heatmap showing the relative abundance of SDMs in LsM and Zs samples. Rows represent individual SDMs, and columns represent samples (LsM1–LsM6, LS1–LS6, Zs1–Zs6); color intensity indicates the normalized abundance of metabolites (blue to red represents low to high abundance), reflecting the distinct accumulation patterns of SDMs between the two potato cultivars; (**B**) Venn diagram of SDMs across the three pairwise comparisons (Zs_vs._LsM, LsM_vs._LS, Zs_vs._LS) in positive (upper panel) and negative (lower panel) ion mode. The diagram quantifies the overlap and uniqueness of SDMs: numbers in overlapping regions indicate shared SDMs between comparisons, and non-overlapping regions represent SDMs specific to a single comparison; (**C**–**E**), Machine learning predictions of the key SDMs between LsM, LS, and Zs. Each panel lists the top 20 metabolites, color intensity indicating relative abundance; (**F**–**H**) Kyoto Encyclopedia of Genes and Genomes (KEGG) pathway enrichment analysis of DAMs of LsM vs. LS vs. Zs (**F**), LsM vs. Zs (**G**), and LsM vs. LS (**H**) comparisons. The x-axis represents -log10(*p*-value) (higher values = more significant enrichment).

The VIP (variable importance in the projection) value is an essential metric that reflects a metabolite’s importance and is used for assessing the SDMs between samples. Its threshold value as a criterion for SDMs was VIP > 1. In the POS mode, 205 SDMs were identified in LsM when compared with Zs (159 were more abundant and 46 were less abundant), whereas 58 SDMs were identified in the NEG mode (46 were more abundant and 12 were less abundant), with a total of 263 SDMs between LsM and Zs (Figure 4A,B and Supplementary Table S8). There are 189 SDMs in the POS mode that were identified in LS when compared with Zs (125 were more abundant and 64 were less abundant), whereas 51 SDMs were identified in the NEG mode (31 were more abundant and 20 were less abundant), with a total of 240 SDMs between LS and Zs (Figure 4A,B and Supplementary Table S8). And 187 SDMs in the POS mode were identified in LS when compared with LsM (80 were more abundant and 107 were less abundant), whereas 50 SDMs were identified in the NEG mode (16 were more abundant and 34 were less abundant), with a total of 237 SDMs between LS and LsM (Figure 4A,B and Supplementary Table S8). When comparing all three group samples (LsM, LS, and Zs), there are 380 SDMs that were identified in total—298 SDMs in the POS mode and 82 SDMs in the NEG mode. Correlation analysis of SDMs revealed a high correlation between SDMs (Figure 4A,B and Supplementary Table S8).

To predict the key metabolites that contribute to the flavor of the Lushan Russet potato, machine learning was used for the SDMs between LsM, LS, and Zs. The results speculated the top 20 metabolites that may play important roles in different flavors between LsM, LS, and Zs (Zs vs. LsM vs. LS), between LsM and Zs (Zs vs. LsM), and within Lushan Russet potatoes (LsM vs. LS) (Figure 4C–E). More metabolites were highly accumulated in Lushan Russet potatoes (LsM and LS) than in Zs, with the most notable difference observed in the “Zs vs. LsM” comparison (Figure 4D). This accumulation pattern may explain the flavor variations between Lushan Russet potatoes and Zs potatoes, and these metabolites are likely to play essential roles in shaping the flavor of Lushan Russet potatoes. There are also metabolites that have different accumulation within LsM and LS Lushan Russet potatoes. These machine learning-derived results predicted the metabolites that predominantly contribute to the flavor variation between LsM and LS (Figure 4E).

KEGG enrichment analysis of SDMs suggested that the top 20 enriched pathways, these pathways are mostly related to metabolism, except for ABC transporters and Aminoacyl-tRNA biosynthesis, which belong to environmental information processing and genetic information processing, respectively (Figure 4F–H). This data may indicate that the metabolism process is the main reason for the metabolic differences between the Lushan Russet potato and Zs (Figure 4F–G). Moreover, besides metabolism, environmental information processing and genetic information processing may also play roles in the differences within Lushan Russet potatoes, therefore contributing their flavor variation, although they share the same genetic background (Figure 4H).

Taken together, our results demonstrate that more abundant SDMs in Lushan Russet potato are more abundant than less abundant SDMs, which suggests that the more abundant metabolites in Lushan Russet potato may contribute to the flavor and taste in comparison to Zs. Lushan Russet potato shows differences in taste and flavor when grown at different altitudes, which is also reflected in the variations of their metabolites.

### 3.5. Endophytic Microbiome Diversity in Lushan Russet Potato Tubers Varies Across Different Growing Positions

In order to detect microbiome composition diversity in Lushan Russet potato tubers across different growing positions, we profiled bacterial and fungal endophytic communities of LsM and LS tubers using DNA amplicon sequencing of 16S rRNA gene and the internal transcribed spacer (ITS) of nuclear rRNA, respectively.

We obtained 3,000,147 and 4,583,280 valid sequences from 16 samples belonging to two groups of potato tubers (LsM and LS, with 8 sample replicates per group), for the 16S rRNA and ITS assays, respectively. After host sequence filtering, 641,505 and 109,483 pure reads were left for further analysis (Supplementary Tables S9 and S10). Finally, we generatied 4074 ASVs (amplicon sequence variants) for bacterial community assays and 311 ASVs for fungal community assays (Supplementary Tables S11 and S12). There was no significant difference of the bacterial community’s (Supplementary Table S13) and fungal community’s (Supplementary Table S14) alpha diversity between different tubers, as shown by the Chao1, Shannon, and Simpson indexes, indicating that the bacterial and fungal endophytes have no significant difference in richness.

**Figure 5:**
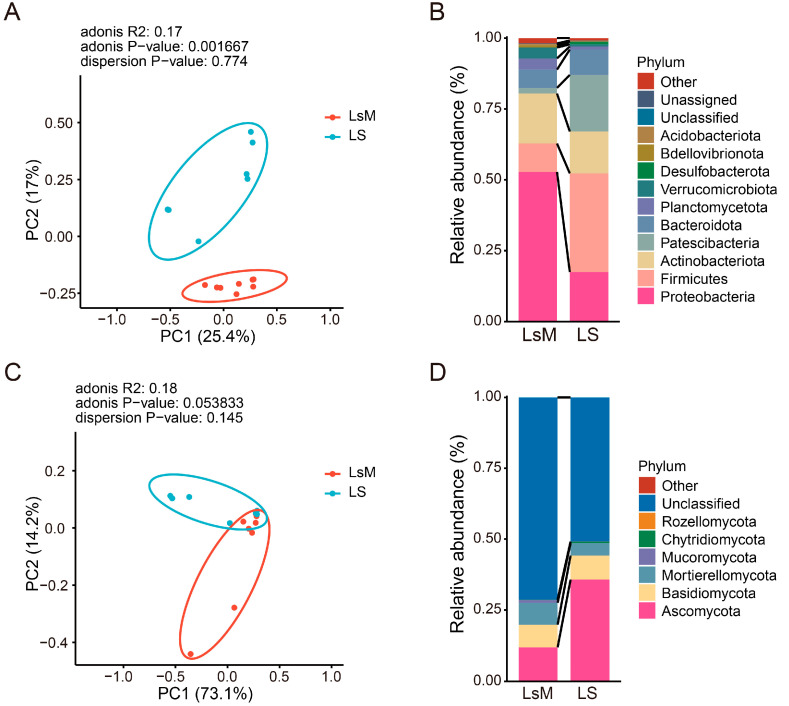
Beta diversity and phylum-level relative abundance of endophytic bacteria and fungi communities in LsM and LS potato tubers. (**A**) Principal Coordinates Analysis (PCoA) plot of endophytic bacteria based on the Bray–Curtis distance. (**B**) Phylum-level relative abundance of endophytic bacteria. (**C**) Principal Coordinates Analysis (PCoA) plot of endophytic fungi based on the Bray–Curtis distance. (**D**) Phylum-level relative abundance of endophytic fungi.

The beta diversity of bacterial and fungal endophytes was analyzed using principal coordinates analysis (PCoA) based on the Bray–Curtis distance, and PCoA plots were generated using the first two principal coordinates (Figure 5A,C). Potato tubers from two groups (LsM and LS) were basically clustered into two groups in both bacterial and fungal endophytes, especially for bacterial endophytes assays (Figure 5A,C). The first principal coordinate (PC1) accounted for 25.4% of the total variation in bacterial compositional dissimilarity among samples, while the second principal coordinate (PC2) explained an additional 17% of the variance. Together, these two primary axes captured 42.4% of the overall bacterial endophytic community variation (Figure 5A) (adonis R^2^: 0.17, adonis *p*-value: 0.001667, dispersion *p*-value: 0.774). For the fungal endophytic community, the first principal component explained 73.1% of the variation, whereas the second principal component explained 14.2% of the variation (adonis R2: 0.18, adonis *p*−value: 0.053833, dispersion *p*-value: 0.145) (Figure 5C). These data may suggest that the largest source of variation in microbial communities is introduced by difference in potato tubers.

Abundance profile analyses identified groups of bacteria and fungi consistently enriched in LsM and LS (Figure 5B,D, Supplementary Tables S15 and S16). Our data show that phyla *Proteobacteria*, *Firmicutes*, *Actinobacteriota*, and *Patescibacteria* were the dominant bacterial phyla, and *Proteobacteria* and *Actinobacteriota* were the most dominant phylum in LsM group, with 52.8% and 17.6% abundance, respectively, but those bacterial phyla were depleted to 17.5% and 14.8% in the LS group, whereas phyla *Firmicutes* and *Patescibacteria* were increased to 34.% and 20% from 10% and 2% in LS group compare with group LsM (Figure 5B, Supplementary Table S15). Previous studies have reported that *Firmicutes*, *Proteobacteria,* and *Bacteroidetes* are the dominant microbial phyla in tuber tissues, which is consistent with our data [35]. However, certain discrepancies were also observed, potentially attributable to the different physiological states of the tubers examined. Specifically, the tubers in those studies were sampled at the germination stage, whereas the experimental materials in our work were freshly harvested tubers. Moreover, our data revealed striking variations in endophytic microbial compositions among tubers collected from different cultivation sites. Collectively, these findings highlight that the endophytic microbial communities of tubers are strongly influenced by both the host’s physiological status and the growing environment [35,36,37].

Due to the lack of comprehensive reference sequences in existing fungal databases, a large proportion of fungal ASVs obtained from sequencing failed to be assigned to clear taxonomic ranks and were thus classified as “Unclassified”. Among the fungal ASVs that successfully obtained taxonomic annotations, they were affiliated with six major phyla, namely *Ascomycota*, *Basidiomycota*, *Mortierellomycota*, *Mucoromycota*, *Chytridiomycota*, and *Rozellomycota* (Figure 5D, Supplementary Table S16). Notably, *Ascomycota* was the most abundant fungal phylum across the annotated community. Its relative abundance exhibited significant variation between the two sample groups: in the LsM group, the relative abundance of *Ascomycota* was 11.8%, while in the LS group, it increased to 35.7% (Figure 5D, Supplementary Table S16). Certain fungal taxa exhibit higher phylum—level abundances in LS compared to LsM. However, within the same phylum, the abundances of some microbial groups at different taxonomic levels can be reversed. For example, *Cladosporium*, a genus of fungi belonging to the phylum *Ascomycota*, shows an approximately three-fold higher abundance of *Ascomycota* in LS than in LsM. At the order, family, and genus levels (*Cladosporiales*), the abundance in LsM is nearly twice that in LS (Supplementary Table S16).

From our data, after host sequence removal, the proportion of clean reads (usable reads) relative to the total reads is notably low in endophytic microorganism analysis. For endophytic bacteria, the average percentage of usable reads post—host DNA depletion is approximately 21% (Supplementary Table S9). In contrast, the number of usable reads for endophytic fungi analysis is even more limited for certain samples; the proportion of usable reads drops to less than 1% of the total reads (Supplementary Table S10). Despite the fact that we have increased the sequencing data volume, the amount of informative data obtained remains limited. This observation suggests that simply scaling up the data size may not effectively address the underlying constraints (e.g., persistent host DNA interference, low biomass of target endophytes) that restrict the acquisition of meaningful biological information for subsequent endophytic community analysis. Maybe the application of new technologies in the future, such as the absolute quantification of microorganisms, will be able to reveal more information [38,39]. In fungal diversity research, especially studies leveraging high-throughput amplicon sequencing (e.g., ITS) to characterize ASVs or OTUs, incomplete taxonomic reference databases remain a critical bottleneck limiting accurate taxonomic annotation and ecological interpretation [22]. As observed in this study, a large proportion of fungal ASVs were classified as “Unclassified,” which may be ecologically significant taxa, distorting estimates of community composition and hindering comparisons of fungal assemblages across experimental treatments.

### 3.6. Key Endophytic Microbial Taxa Predicted by LEfSe and Random Forest Analyses

Linear discriminant analysis effect size (LEfSe) was performed to identify bacterial and fungal taxa with significant differential abundance between the LsM and LS sample groups, and to evaluate their discriminative power. The analysis was conducted with a strict linear discriminant analysis (LDA) threshold set to 4.0 and 2.0 for bacterial and fungal, respectively.

**Figure 6:**
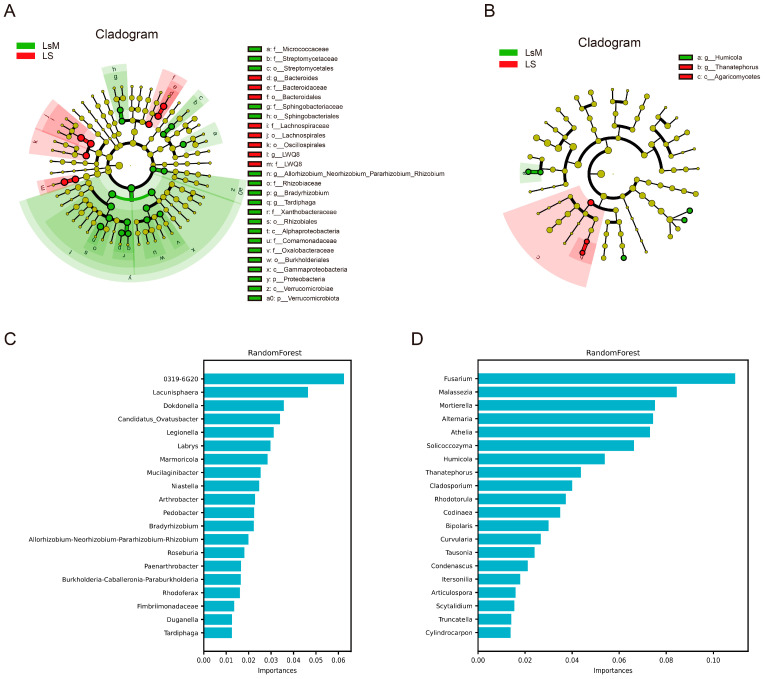
LEFSe and Random Forest analysis of endophytic microbial communities in LsM and LS potato tubers. (**A**,**B**) LEFSe cladograms of endophytic microbial taxa. These two cladograms visualize the hierarchical taxonomic classification of endophytic bacteria (**A**) and fungi (**B**) that exhibit significant differences between LsM and LS potato tubers, covering multiple taxonomic ranks (phylum [p], class [c], order [o], family [f], genus [g]). (**C**,**D**) Random Forest analysis of endophytic taxon importance. Calculated by the Random Forest algorithm to quantify the relative importance of the differential endophytic taxa in distinguishing between LsM and LS potato tubers. Bacteria (**C**) and fungi (**D**).

As visualized in the cladograms (Figure 6A,B), distinct sets of discriminatory taxa were enriched in each group. For bacteria, a hierarchical array of differential taxa was identified across multiple ranks: at the phylum level, p_*Proteobacteria* and p_*Verrucomicrobiota* were prominent discriminants; at the class level, c_*Alphaproteobacteria*, c_*Gammaproteobacteria*, and c_*Verrucomicrobiae* contributed to group separation; at the order level, o_*Bacteroidales*, o_*Sphingobacteriales*, o_*Lachnospirales*, o_*Oscillospirales*, o_*Rhizobiales*, and o_*Burkholderiales* were biomarkers; at the family level, f_*Bacteroidaceae*, f_*Sphingobacteriaceae*, f_*Lachnospiraceae*, f_*LWQ8*, f_*Rhizobiaceae*, f_*Xanthobacteraceae*, f_*Comamonadaceae*, and f_*Oxalobacteraceae* exhibited significant differential enrichment; and at the genus level, g_*LWQ8*, g_*Allorhizobium_Neorhizobium_Pararhizobium_Rhizobium*, g_*Bradyrhizobium*, and g_*Tardiphaga* were confirmed as high-confidence bacterial biomarkers (Figure 6A). For fungi, g_*Humicola*, g_*Thanatephorus*, and c_*Agaricomycetes* emerged as core fungal biomarkers, with clear taxonomic clustering associated with either the LsM or LS group. These LEfSe identified taxa collectively delineated the taxonomic divergence of microbial communities between the two sample groups (Figure 6B).

The Random Forest algorithm was further applied to the microbial abundance to quantify feature importance and validate key discriminatory taxa (Figure 6C,D). Fungal taxa, such as g_*Humicola* and g_*Thanatephorus* (highlighted by LEfSe), exhibited notable importance values (Figure 6B,D). *Cladosporium*, another fungal taxon clearly presented in the feature importance analysis, also emerged as potential significantly in distinguishing sample groups (Figure 6D). For bacterial taxa, those including g_*Allorhizobium_Neorhizobium_Pararhizobium_Rhizobium*, g_*Bradyrhizobium*, and representatives from families like f_*Bacteroidaceae* and f_*Rhizobiaceae* also ranked among the top contributors to predictive power (Figure 6C). Specifically, taxa such as g_*Humicola* and p_*Proteobacteria* affiliated ASVs displayed relatively higher importance scores (~0.06), indicating their critical role in distinguishing LsM and LS samples (Figure 6C).

This convergence between LEfSe -derived differential taxa and Random Forest- identified high-importance features confirms the ecological relevance of these microbial groups, validating them as key biomarkers for characterizing the distinct microbial community structures of the LsM and LS sample groups, and these data were consistent with the metabolomic data in this study. This mutual correspondence may imply potential functional associations between the shifts in endophytic assemblages and the alterations in the host’s metabolic pathways, thereby providing complementary evidence for interpreting the flavor differences in Lushan Russet potato under distinct high-altitude environmental conditions. Endophytic communities showed variations with increasing altitude, which play pivotal roles in nutrient acquisition, stress tolerance, and secondary metabolism regulation of host plants, finally influencing the potato flavor.

## 4. Conclusions

Lushan Russet potato is a valuable local cultivar distinguished by its unique flavor, but the chemical and biological mechanisms underlying this flavor, as well as its degradation at lower altitudes, have remained unclear. This study systematically compared LsM, its low-altitude-grown variant (LS), and the reference cultivar Zhongshu 5 (Zs) in terms of nutritional components and metabolite profiles, and further analyzed the diversity of the endophytic microbiome in LsM and LS. Results showed LsM and LS possess superior nutritional quality compared to Zs (with higher dry matter, starch, potassium, vitamin C, amino acids, fat, and sugar). Genotypes drove LsM-Zs metabolic/flavor differences (263 SDMs, mostly more abundant in LsM in metabolic pathways), while altitude modulated LsM-LS metabolism (237 SDMs). Endophytic microbiomes (with distinct beta diversity and taxon shifts like more Firmicutes/Ascomycota in LS) play an important role in shaping LsM’s flavor, particularly in response to altitude, supported by microbiome-metabolome correlation. Overall, the Lushan Russet potato’s flavor stems from genetically driven flavor-related nutrient/metabolite accumulation and altitude-induced microbiome shifts. These findings not only fill gaps in our understanding of LsM’s flavor biology but also provide practical insights: for instance, targeted breeding could prioritize the retention of key flavor-related genes, while microbiome manipulation might mitigate flavor deterioration in low-altitude cultivation. Ultimately, this work lays a foundation for optimizing LsM’s quality and promoting its sustainable industrial development.

## Data Availability

The raw data of the amplicon sequencing have been deposited in the China National Center for Bioinformation (https://www.cncb.ac.cn), with the accession numbers CRA029784 for bacteria and CRA029782 for fungi. The other original contributions presented in the study are included in the article/Supplementary Material, further inquiries can be directed to the corresponding authors.

## Supplementary Materials

The following supporting information can be downloaded at: https://www.mdpi.com/article/10.3390/foods15010067/s1, Supplementary Figure S1: Sample correlation analysis and hierarchical cluster analysis based on metabolite profiles. Upper panels, Positive ion modes; lower panels, Negative ion modes; Supplementary Figure S2: Volcano plots show the differences between LsM, LS, and Zs potatoes. POS, positive ion modes; NEG and negative ion modes; Supplementary Figure S3: The chemical compositions of LsM, LS, and Zs potato tubers. Upper panels, positive ion modes; lower panels, negative ion modes. Supplementary Table S1: Identified effective peaks in the positive ion mode (POS); Supplementary Table S2: Identified effective peaks in the negative ion mode (NEG); Supplementary Table S3: Source data of Supplementary Figure S1 upper panels; Supplementary Table S4: Source data of Supplementary Figure S1 lower panels; Supplementary Table S5: Source data of Supplementary Figure S2 left panels; Supplementary Table S6: Source data of Supplementary Figure S2 right panels; Supplementary Table S7: Source data of Supplementary Figure S3 upper panels; Supplementary Table S8: Source data of Supplementary Figure S3 lower panels; Supplementary Table S9: Raw reads and clean reads information of bacteria community assays; Supplementary Table S10: Raw reads and clean reads information of fungi community assays; Supplementary Table S11: Relative abundance of endophytic bacterial ASVs in LsM and LS samples; Supplementary Table S12: Relative abundance of endophytic fungal ASVs in LsM and LS samples; Supplementary Table S13: Alpha diversity of endophytic bacterial communities in LsM and LS; Supplementary Table S14: Alpha diversity of endophytic fungal communities in LsM and LS; Supplementary Table S15: Average relative abundance of endophytic bacteria at different taxonomic levels (LsM vs. LS); Supplementary Table S16: Average relative abundance of endophytic fungi at different taxonomic levels (LsM vs. LS).
