# Supplementary figures and images for: Integrative Analysis of Nutritional Components, Differential Metabolites, and Endophytic Microbiota Reveals Flavor Determinants of Lushan Russet Potato
Source: Foods. 2025 Dec 25;15(1):67. doi: 10.3390/foods15010067 (PMC12785464; doi:10.3390/foods15010067)

Supplementary Figure S1

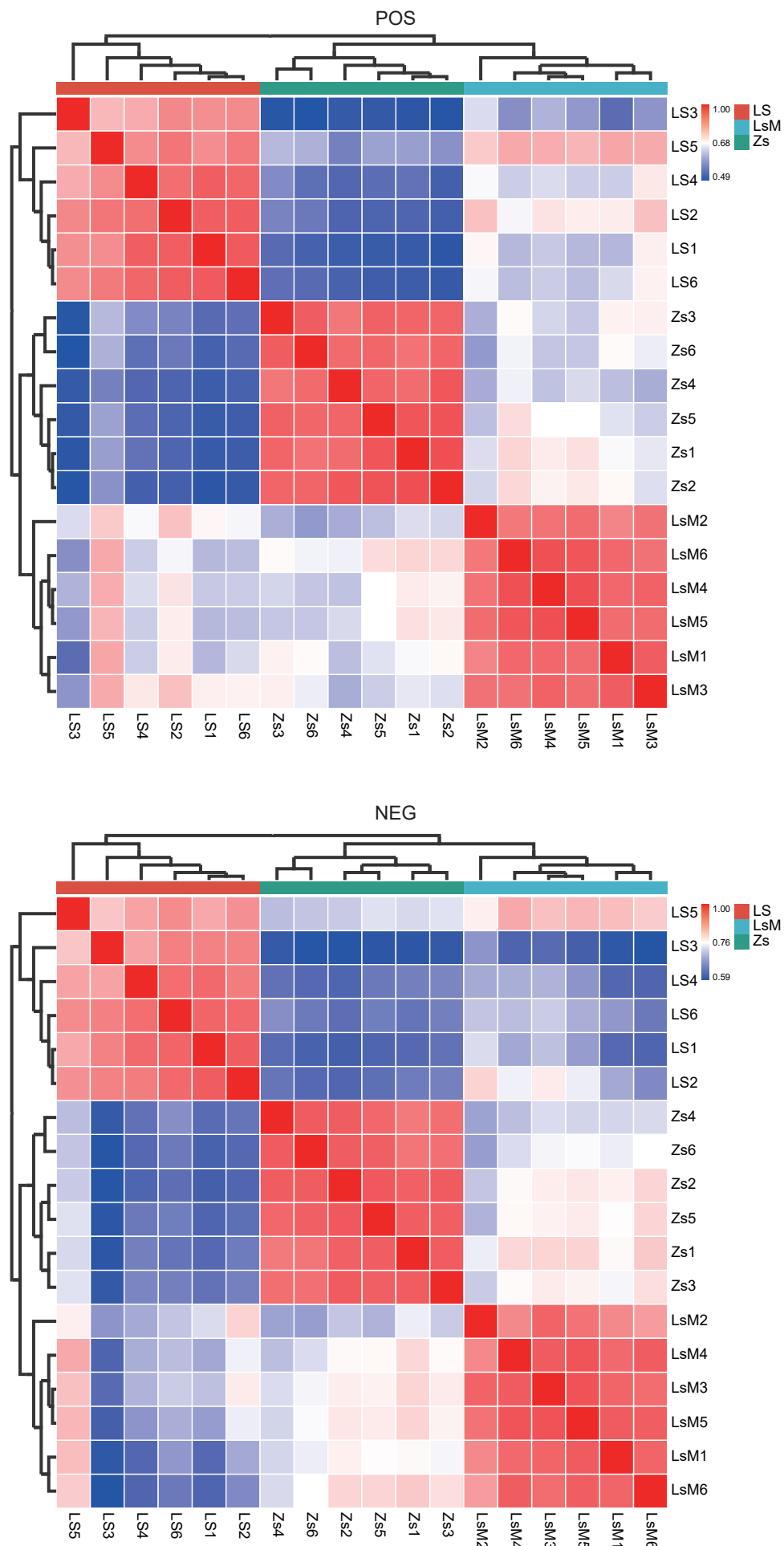

Supplementary Figure S2

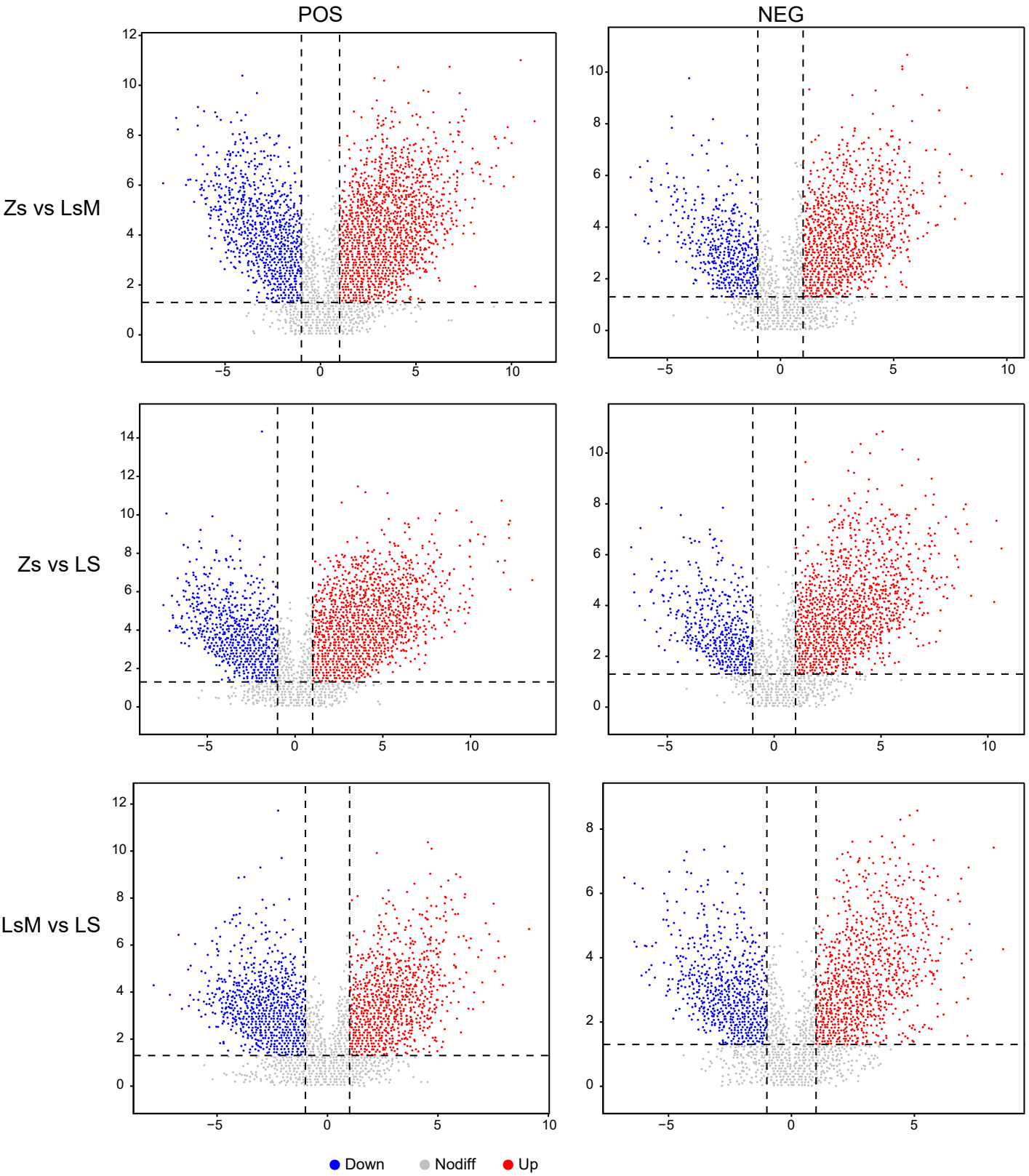

Supplementary Figure S3

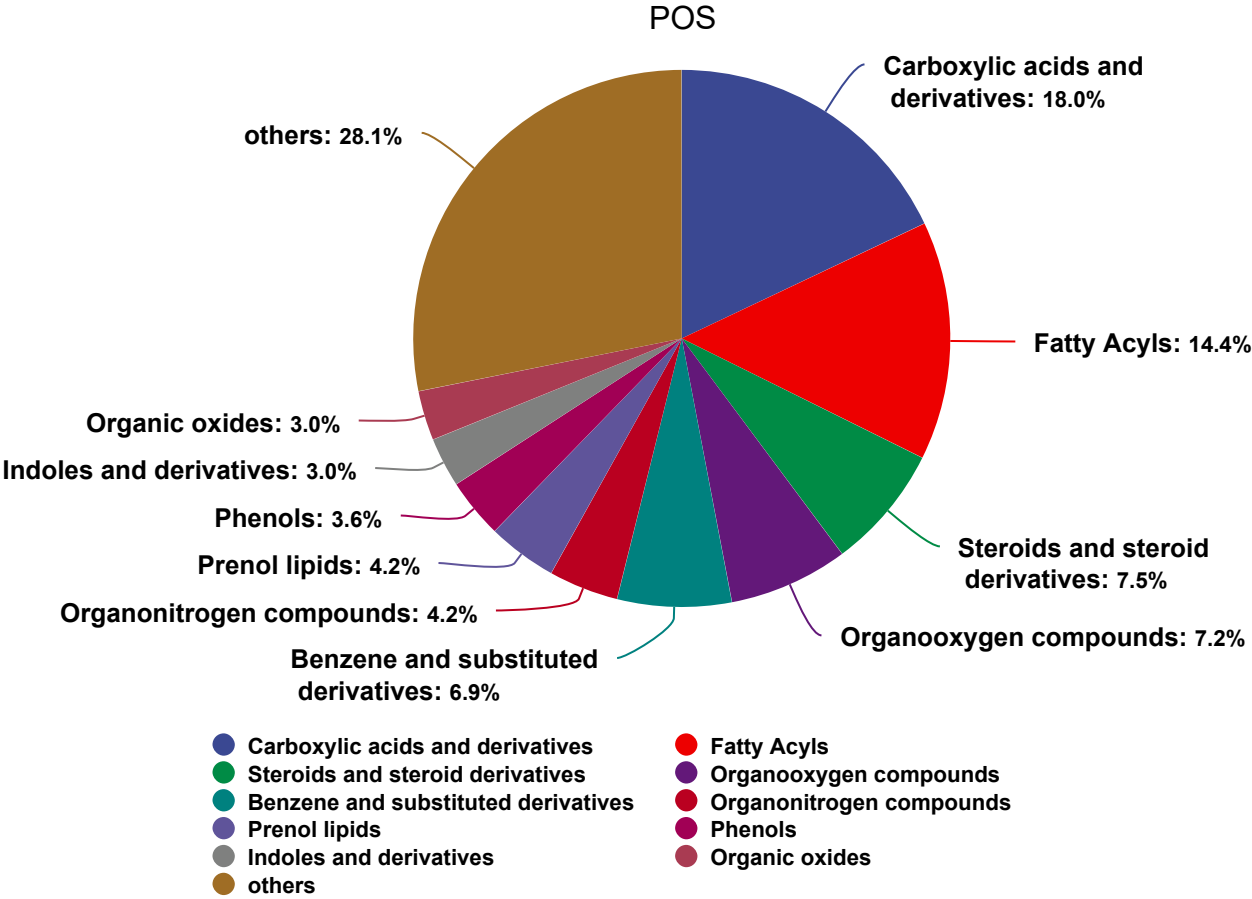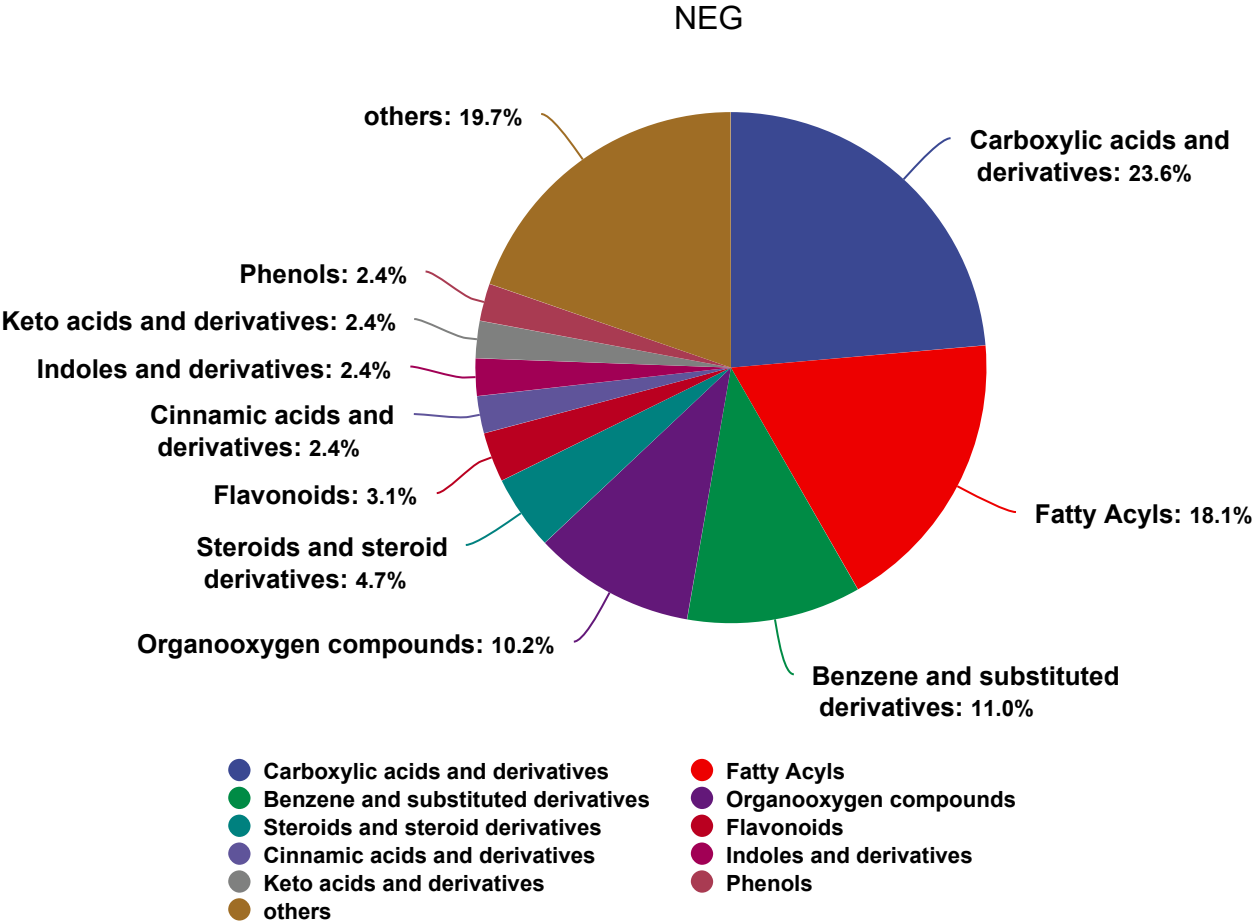

Supplement: Supplementary file 1 [file foods-15-00067-s001.zip › Supplementary Figures.pdf]
